# Cascade Proportional–Integral Control Design and Affordable Instrumentation System for Enhanced Performance of Electrolytic Dry Cells

**DOI:** 10.3390/s24165427

**Published:** 2024-08-22

**Authors:** Saulo N. Matos, Gemírson de Paula dos Reis, Elisângela M. Leal, Robson L. Figueiredo, Thiago A. M. Euzébio, Alan K. Rêgo Segundo

**Affiliations:** 1 Institute of Mathematics and Computer Science, University of São Paulo, São Paulo 05508-220, SP, Brazil; saulo.matos@usp.br; 2 Programa de Pós-Graduação em Instrumentação, Controle e Automação de Processos de Mineração, Universidade Federal de Ouro Preto (UFOP), Ouro Preto 35402-163, MG, Brazil; gemirson.reis@ufop.edu.br (G.d.P.d.R.); alankardek@ufop.edu.br (A.K.R.S.); 3 Department of Mechanical Engineering (DEMEC), Universidade Federal de Ouro Preto (UFOP), Ouro Preto 35402-163, MG, Brazil; elisangelamleal@ufop.edu.br; 4 Program in Mineral Engineering (PPGEM), Universidade Federal de Ouro Preto (UFOP), Ouro Preto 35402-163, MG, Brazil; robsonlage@ufop.edu.br; 5 Helmholtz-Zentrum Dresden-Rossendorf, Institute of Fluid Dynamics, 01328 Dresden, Germany; 6 Virtus-CC, Campina Grande 58429-140, PB, Brazil

**Keywords:** electronic instrumentation, cascade control, proportional integral, electrolysis, hydrogen

## Abstract

In this paper, we present a cost-effective system for monitoring and controlling alkaline electrolyzers, intending to improve hydrogen gas production on a laboratory scale. Our work includes two main innovations. Firstly, we suggest an approach to calibrate a standard air flow meter to accurately measure the flow of hydrogen-rich gas from electrolyzers, improving measurement accuracy while keeping costs low. Secondly, we introduce a unique cascade control method to manage hydrogen-rich gas production in the electrolyzer, ensuring precise control over gas flow rates. By combining affordable, energy-efficient devices with a PI control system, we achieve efficient gas production through electrolysis, replacing manual control approaches. Experimental results confirm the effectiveness of our cascade control method, demonstrating stable operation with minimal errors. These results provide a foundation for further research into control strategies to enhance the performance of electrolytic cells.

## 1. Introduction

With the ongoing threat of global warming and the increasing necessity to tackle environmental issues, developing sustainable energy solutions is crucial. As the global population of 7.7 billion people currently requires 42,043 trillion BTU of energy annually, a figure that is expected to rise continuously, particularly in developing nations, it is projected that global energy demand will increase by 24% by 2040 compared to 2019 [1,2,3]. In this context, renewable energy sources like wind and solar power emerge as promising alternatives, though their intermittent nature necessitates efficient energy management and storage strategies [4,5,6]. Hydrogen, with its impressive energy density and environmental advantages, stands out as a particularly attractive storage medium. Boasting an energy density of 140 MJ/kg, hydrogen surpasses conventional solid fuels by more than twice, making it a potent choice for energy storage [7]. Additionally, hydrogen combustion results in water as the only byproduct, positioning it as an environmentally friendly energy carrier. Given the projected 70% increase in energy consumption in Non-OECD countries between 2018 and 2050 [8], diversifying energy generation by shifting towards renewable sources such as fuel cells, solar cells, photovoltaic cells, supercapacitors, and batteries is crucial for sustaining future generations [1,9,10].

Like many other industries, hydrogen production heavily relies on automation to improve security and lower operational costs [11]. It is crucial to select the right control loops and strategies to achieve maximum efficiency at minimal cost. Equally important is choosing suitable instrumentation to provide controllers with the necessary data for effective operation. Extensive research has been conducted to develop advanced control strategies for hydrogen-rich gas production, such as innovative sensor calibration methods and the use of cascade control systems to enhance performance in research labs and small-scale settings. These advancements aim to enhance the reliability, efficiency, and scalability of hydrogen production processes, making them more suitable for various applications.

In this paper, we present two significant contributions aimed at advancing control methodologies for hydrogen gas production using alkaline electrolyzers. First, we propose a method for calibrating a conventional and cost-effective air flow meter to accurately measure the flow rate of hydrogen-rich gas in electrolyzer outputs. This calibration approach is especially important because of the large price difference between air flow meters and those designed specifically for measuring $H_{2}$ flow. Using the most cost-effective solution, we can incorporate these systems into affordable research projects without sacrificing measurement quality, thus improving control system capabilities. Second, we introduce an innovative control strategy for regulating the production of hydrogen-rich gas within the electrolyzer. This approach involves implementing a cascade control system. The primary control loop is designed to track the gas flow rate against a predefined reference. This is performed by adjusting the current reference within a secondary internal control loop. The secondary loop, in turn, adjusts the Pulse Width Modulation (PWM) duty cycle of a voltage controller to maintain the desired electric current input to the electrolyzer, thus ensuring precise control over $H_{2}$ gas production. This new control structure improves the efficiency and reliability of the electrolyzer operations, leading to better performance in different applications.

## 2. Related Works

This section discusses works that monitor or control hydrogen generator systems. Expensive sensors and actuators are typically used in automatic feedback control studies of hydrogen gas production as demonstrated in a study where industrial sensors costing approximately BRL 1500 were utilized for control purposes [12,13]. Alternatively, our work proposes the development of cost-effective instrumentation and control systems based on a low-cost gas flow sensor and an 8-bit microcontroller. Using a low-cost microflow sensor could reduce the price of flow sensors by 95% [14].

There are various ways to generate different kinds of hydrogen [15]. Hydrogen can be generated through various processes, including steam methane reforming (SMR), coal gasification, biomass gasification, and water electrolysis [15,16,17]. Water electrolysis can be conducted using various devices, including alkaline electrolyzer, dry cells, wet cells, hybrid cells, and polymer electrolyte membrane (PEM) systems [18].

Baltacıoğlu [19] used a PWM signal generated by an Arduino board to control the voltage applied in a dry cell alkaline electrolyzer. The electrolysis gas was used as an additive in a motor, and the electrolyzer’s behavior was monitored. However, it was an open-loop control. Reference [20] proposes an instrumentation system without measuring the gas flow and proposing an automatic control of a dry cell alkaline electrolyzer. The proposed system was tested on a diesel generator, generating a consumption reduction of 3.25%.

Reference [21] developed a simulation to model and control a polymer electrolyte membrane (PEM) electrolyzer. The system was identified using a white box method, and a nonlinear Model Predictive Control algorithm was used to minimize the cost of producing hydrogen gas. A predictive control was also used in [22] in an alkaline electrolyzer. Additionally, some papers in the literature use the Hammerstein identification technique to model the electrolyzer and MPC control [12,13]. Cervantes-Bobadilla et al. [12] also used the hydrogen-rich gas as an additive to gasoline; there was an improvement in thermal and combustion efficiency of 4% and 0.6%, respectively, and an increase in power of 545 W, reducing the fossil fuel use.

Reference [23] performed a simulation of a dual loop cascade control of a PEM electrolyzer. The PI controls the current and voltage across the load. Despite the fact that it is a PI cascade control, it differentiates of our paper in the variables controlled in each loop. Our paper has an internal current loop and an external hydrogen mass flow loop.

Ruomei et al. [24] proposed a third-order time-delay thermal model and two controllers: a current feed-forward PID controller and a model predictive controller (MPC). The experimental results demonstrate significant improvements in temperature control, reducing overshoot and increasing efficiency by allowing higher temperature set-points.

Folgado et al. [25] discussed safety measures for hydrogen generation using a Proton Exchange Membrane Electrolyzer (PEMEL) integrated into a smart microgrid powered by renewable energy. It details the development of sensor-based algorithms to ensure the safe and stable operation of the PEMEL by monitoring parameters like temperature, pressure, and water availability. The system’s implementation involves a programmable logic controller managing the electrolyzer’s complete cycle to avoid equipment degradation and malfunctions, with the experimental results demonstrating effective real-time operation. In [26], a data acquisition and monitoring system for PEM hydrogen generators is presented. It leverages the Industrial Internet of Things (IIoT) to enhance performance and reliability. The system integrates various industrial components, including sensors, a PLC for data acquisition, and a middleware layer for data processing and storage. All of these components are accessible through a web-based interface.

Reference [27] proposed two control strategies that mitigate the cross-contamination of $H_{2}$ and $O_{2}$ in a high-pressure alkaline electrolyzer. It suggests a PI control and others based on optimal control tools. Our work used a combination of grey and black boxes for system identification. Using the Process Reaction Curve method, a grey box approach was employed to model the flow control process. Furthermore, we used the black box method to model the current control process through MATLAB R2022a system identification tool.

The relevance of the indicated development is high due to its application in hydrogen production. Our contribution, the proportional–integral (PI) cascade controller, is a breakthrough in this field, specifically in the context of alkaline electrolyzers. Accordingly, using different approaches to dynamic modeling, controlling, and monitoring systems in this research field can be considered an important step in obtaining cleaner and more economical energy. This work uses a low-cost system, including the gas flow sensor, for feedback cascade control of the current and the flow of hydrogen gas applied to an electrolytic cell. Table 1 summarizes different works focused on electrolyzer control and monitoring.

## 3. Materials

**Figure 1:**
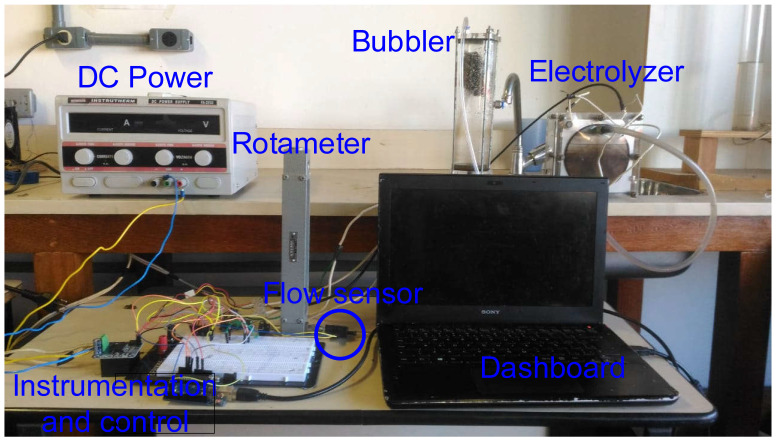
Experiment setup for electrolytic cell.

For this study, we focused our experimental setup on a dedicated test bench with key components as shown in Figure 1. The electrolysis processes were carried out in a rectangular plate electrolytic cell, using a single-channel FA-2030 Instrutherm Digital DC power supply that can deliver up to 32 volts and 20 amperes. Our setup was enhanced with a control and instrumentation system managed by an Arduino UNO R3 board, which received power from a USB supply connected to a personal computer. Renowned for its versatility, the Arduino allows precise control over experimental parameters, considering the project’s size and complexity.

The electrolyte utilized in the electrolyzer consisted of potassium hydroxide (KOH). To produce the necessary electrolysis gas, a solution was prepared with 20 g of potassium hydroxide per liter of distilled water [14,19]. Reference [28] demonstrated that using an electrolyte containing 20–30 g/L of potassium hydroxide (KOH) and applying 10 A of current yielded optimal results. Moreover, our experimental setup included a bubbler to optimize the electrolysis process by separating water particles from the gas product. Gravity-assisted fluid circulation within the bubbler facilitated efficient gas–liquid separation as described by Miyamoto et al. [29].

**Figure 2:**
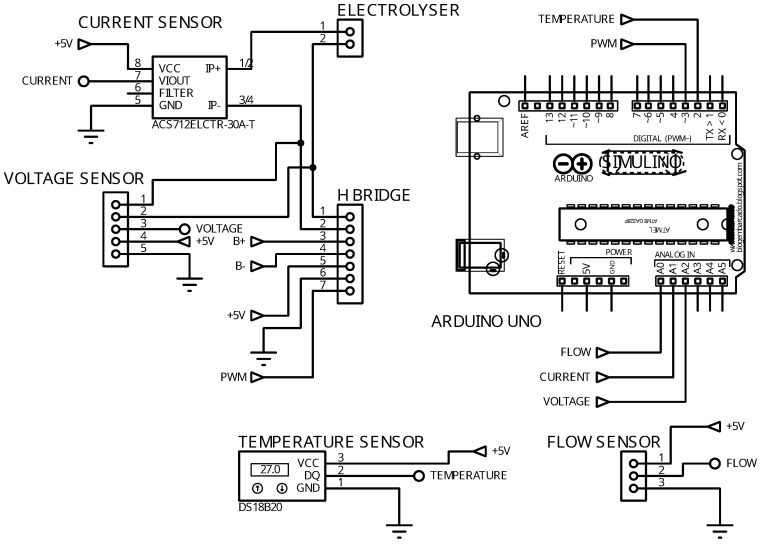
Schematic diagram of instrumentation and control system.

The Arduino was used to collect and analyze sensor data from the electrolytic cell, allowing the real-time monitoring of important variables like electric current, voltage, temperature, and the mass flow of electrolysis gas. Sensor readings were taken every 500 ms, a suitable frequency for applications with limited computational resources. Additionally, the Arduino ran proportional–integral (PI) control algorithms to manage the electrolysis process. Pulse Width Modulation (PWM) was applied to control the voltage supplied to the electrolytic cell by generating voltage pulses of varying durations from a constant power source, effectively adjusting the average voltage to regulate electrical power. By using an Arduino microcontroller, a 25 kHz frequency square wave was created to quickly switch a MOSFET, enabling precise voltage control by adjusting the wave’s duty cycle. For this purpose, a BTS7960 H-bridge MOSFET component was used, capable of handling up to 43 A of current. The H-bridge design includes two inputs for the PWM signal, each for a specific current direction. As control in only one direction was needed, the PWM signal from digital pin 3 of the Arduino was connected to one of the H-bridge terminals. A freewheeling diode was added parallel to the load to protect the voltage control system. The schematic diagram of the instrumentation and control system is shown in Figure 2.

The electric current in the electrolytic cell was measured using the ACS712 current sensor. This sensor detects magnetic fields created by current flow through the Hall effect principle. It was connected to the analog pin 1 of the Arduino, providing a voltage output proportional to the current detected. The sensor has a resolution of 18.5 mA and an uncertainty of ±1.5% of the full scale within its operational range of ±30 A. Voltage measurements were conducted with a 0 V to 25 V voltage divider board, which operates based on voltage division. This setup allowed for precise voltage measurements with a resolution of 4.89 mV, utilizing the Arduino’s analog-to-digital converter (ADC) with a measurement range of 0–5 volts and a 10-bit resolution for analog measurements. Temperature measurements were facilitated by the DS18B20 sensor positioned on the electrolytic cell’s rectangular plates. Operating within the temperature range of −55 °C to 125 °C, this sensor provided accurate readings with an accuracy of ±0.5 °C over the range of −10 °C to 85 °C and a resolution of 0.0625 °C [30,31].

Gas flow measurements were taken using the affordable Winsen F1012 sensor, known for its reliability and quick response time. This sensor uses temperature changes to detect gas flow, providing accurate readings with a maximum error of 2.5% within its range of 0 to 2000 cm^3^/min at the outlet of the electrolytic cell [32,33,34]. The F1012 flow sensor operates on the principle of thermal mass flow sensing. The sensor utilizes a miniature heating element and temperature sensors arranged on a thin film substrate at its core. When fluid flows through the sensor, it causes a change in the temperature distribution around the heating element. The sensor maintains the heating element at a constant temperature above the fluid’s. As the fluid flow increases, it carries away more heat, requiring more power to maintain the element’s temperature. This power requirement is directly proportional to the mass flow rate of the fluid. The sensor’s electronics measure this power consumption and convert it into an electrical signal corresponding to the flow rate. These sensors have proven to be reliable and effective in various fields, widely used in research and practical applications [30,31,32,35,36]. The Winsen F1012, along with the current and voltage sensors in this study, has been previously used in similar research settings [36]. Additionally, the DS18B20 temperature sensor, commonly found in Internet of Things (IoT) setups, was also utilized [30,31,35].

**Figure 3:**
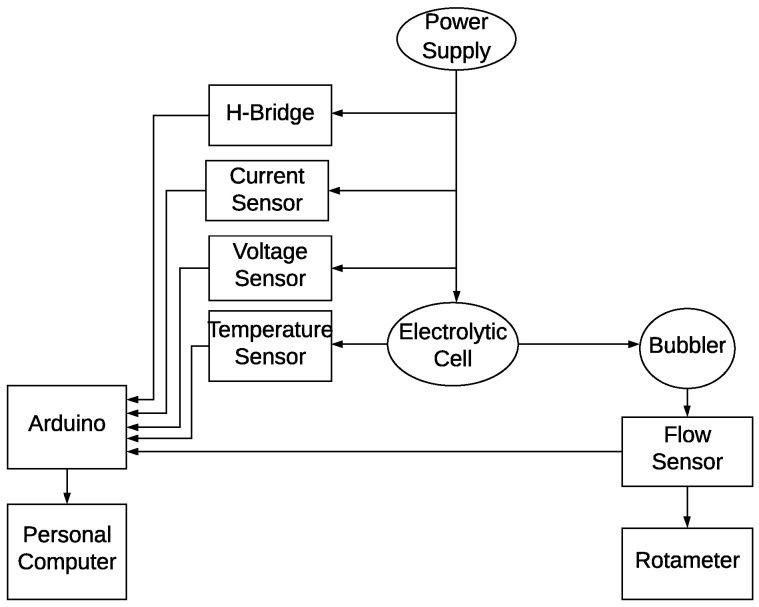
Measurement chain.

Each electronic device was chosen for its cost effectiveness and suitability to meet the electrolyzer’s operational needs. A comprehensive overview of the measurement chain and calibration process is provided in Figure 3.

## 4. Methods

This section outlines the proposed methods in this paper. The first method involves calibrating the gas flow sensor Winsen F1012 for hydrogen-rich gas applications. The second method explains the design and tuning of controllers for the system to automatically determine the gas flow rate production value in the setup.

### 4.1. Gas Flow Sensor Calibration

The microflow sensor was calibrated using a rotameter with a measurement uncertainty of 0.1 g/h. This device operates based on the balance of weight, drag, and thrust forces acting on a float, usually made of stainless steel AISI 316 or PTFE, which moves as the gas flow within its glass tubing increases.

In our laboratory, we carefully calibrated the rotameter to measure the mass flow rate of hydrogen gas accurately. This step is crucial because different gases exhibit diverse properties, such as density and viscosity. We used the Japsin Instrumentation (Mumbai, India) acrylic tube hydrogen rotameter, which has a 1/4” BSP connection, a control valve, and a maximum pressure of 10 kg per square centimeter. The calibration process for a rotameter involves a systematic approach to guarantee accurate and dependable fluid flow measurements.

Adjusting the flow through the rotameter was achieved using a control valve to guarantee accuracy in the readings obtained. These readings were then compared with those from a standard reference flowmeter across various flow rates within the rotameter’s operational range. Subsequently, data were collected, analyzed, and utilized to compute the correction values. These correction values were then applied to adjust the rotameter readings accordingly. The accuracy of these adjustments was validated by comparing the adjusted readings with those from the reference flowmeter at different flow rates. The entire calibration process was thoroughly documented, including the procedures, data collected, and any adjustments made.

Great care was taken to specify the experimental conditions necessary for validating a volumetric flow sensor’s functionality as a mass flow sensor. Recognizing the importance of precision, the flow sensor underwent thorough calibration, with experiments conducted under various environmental conditions.

Calibrating the Winsen F1012 sensor was crucial to ensure accurate measurement of the electrolysis gas flow. The calibration process was conducted simultaneously between the sensor needing calibration and a standard or reference calibration system [37]. During the calibration procedure for the F1012 flow sensor, the rotameter was used as the reference instrument. Both sensors were connected one after the other to the gas output of the electrolytic cell as shown in Figure 1.

The bubbler functioned to protect the flow sensor by limiting the moisture in the output of the dry cell. Placing the bubbler before the sensor and between the dry cell output and the flow sensor effectively prevented water condensation during the experiment.

The calibration procedure involved gradually increasing the electric current from 4 to 10 A, adjusting it in 0.5 A increments, and recording the gas flow sensor’s ADC readings. The ADC readings of the Winsen F1012 were compared with the mass flow measured by the rotameter in g/h. We used linear regression analysis to create the sensor flow calibration equation, which we then integrated into the Arduino code for real-time calibration and measurement.

### 4.2. Control Design

We designed a controller to automatically adjust the flow rate of the hydrogen-rich gas generated by the electrolytic cell. The user can set the desired gas flow within a suitable range, and the controller will then work to maintain the measured flow rate at this set value.

**Figure 4:**
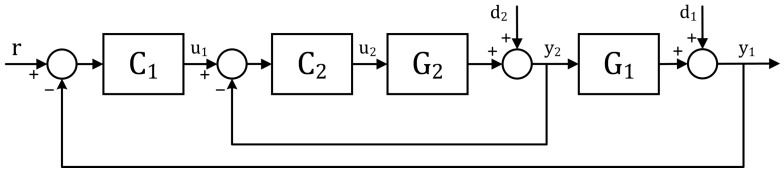
Block diagram for a cascade control approach. Adapted from [38].

Among the various control strategies available for this task, we opted to use a cascade approach with two control loops illustrated in Figure 4. The first loop, known as the external loop, features a proportional–integral (PI) controller ($C_{1}$) that takes the difference between the desired gas flow rate and the actual gas flow rate as input. The output ($u_{1}$) of this controller sets the reference electric current value for the internal loop. The internal loop comprises a PI controller ($C_{2}$) that determines the PWM duty cycle. The transfer functions $G_{1}$ and $G_{2}$ represent the dynamics of the controlled process: $G_{1}$ relates the gas flow rate to the current in the electrolyser, while $G_{2}$ links current to the PWM duty cycle. The choice of a cascade strategy aims to achieve rapid response and minimize the impact of nonmeasured disturbances ($d_{1}$ and $d_{2}$) [38]. Notably, this marks the first instance in the literature where an electric current loop was used to control gas production. Subsequent sections will delve into the specific characteristics of both loops.

#### 4.2.1. Inner Loop—Electric Current

**Figure 5:**
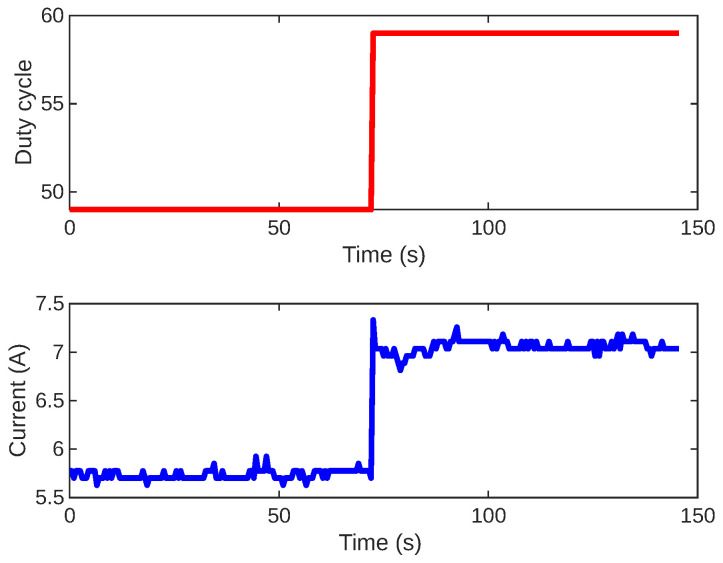
Open-loop step response for the current control system. The PWM duty cycle step is shown in red, and the current response is in blue.

The initial step involves identifying how the inner system behaves by determining the parameters of the transfer function $G_{2}$. This model will be used as a foundation for establishing the PI control parameters of $C_{2}$. To achieve this, we adjusted the duty cycle of the PWM module in increments of 10, ranging from 0 to 79. It is worth noting that this range was attainable because we configured the Arduino’s PWM frequency to 25 kHz [39]. Increasing the PWM frequency serves the purpose of reducing the output ripple and minimizing electromagnetic interference, which is particularly beneficial for delicate applications like current measurement using the Hall effect and achieving quicker response times. In Figure 5, the graph illustrates the current response to a duty cycle change from 49 to 59.

Based on the several step tests conducted in the current loop, we can define the model structure of this system. From Figure 5, it is evident that the system exhibits a rapid response, a gain different from one, and negligible time delay. Therefore, we can express the model structure as follows:

(1) $$G_{2}\left(s\right)=\frac{k_{2}}{\tau_{2}s+1},$$

where $k_{2}$ represents the gain, and $\tau_{2}$ denotes the time constant of the process. We propose to tune the PI controller $C_{2}$ using the identified parameters with the Simple Internal Model Control (SIMC) [40,41,42].

#### 4.2.2. Outer Loop—Gas Flow

**Figure 6:**
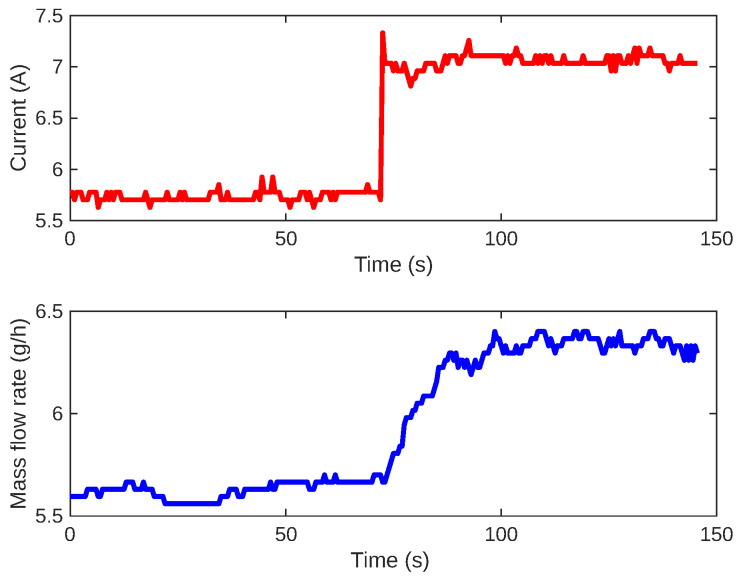
Open-loop step response for the gas flow and current control system. The current is shown in red, and the mass flow rate response is shown in blue.

Similar to the inner loop procedure, we adjust the current incrementally to analyze how the gas flow rate reacts. These adjustments to the input are made in steps. Figure 6 shows a typical reaction curve for this system.

Based on the reaction curve, we define the model structure as a first-order plus time delay as follows:

(2) $$G_{1}\left(s\right)=\frac{k_{1}}{\tau_{1}s+1}e^{-sL_{1}},$$

where $k_{1}$ is the direct gain, $L_{1}$ is the time delay, and $tau_{s}$ is the time constant. Again, these values will be used for tuning the PI controller $C_{1}$ following the rules of the SIMC method.

## 5. Results and Discussion

This section is divided into two parts. First, it details the calibration of the flow sensor for accurate measurement of hydrogen-rich gas. Second, it explains the tuning process of the proposed cascade controller for optimal performance.

### 5.1. Flow Sensor Calibration

Mass flow meters that utilize thermal mass principles calculate fluid flow rates through heat transfer via convection. The convection rate is influenced by the properties of the fluid, underscoring the importance of calibrating thermal dispersion mass flow meters for particular uses. Since each fluid has distinct properties, it is not recommended to use a device calibrated for one gas with a different gas without proper adjustments. The cost-effective F1012 sensor, similar to numerous thermal flow rate sensors, comes precalibrated for air, nitrogen, oxygen, or methane but not for hydrogen gas mixtures. Therefore, recalibration is essential for applications involving hydrogen.

According to the manufacturer, the sensor used in this study comes calibrated for air, measuring from 0 to 2000 cm^3^/min. However, this range may vary when using a gas with different thermal properties, like hydrogen. Hydrogen has higher heat transfer capabilities than air, needing less energy to keep the temperature difference constant. This can be explained by the fundamental heat transfer equation, which shows how the thermal properties of a fluid affect the energy required to maintain a temperature difference:

(3) $$Q=\dot{m}C\Delta T,$$

where *Q* is the heat transfer rate (J/s), $\dot{m}$ is the mass flow rate of the fluid (kg/s), *C* is the specific heat capacity of the fluid ($\frac{J}{\mathrm{kg}\;\cdot {}^{\circ}C}$), and ΔT is the temperature difference between the heated element and the temperature sensors (°C). Due to the thermal properties of hydrogen, the ADC range narrowed significantly (from 210 to 280).

**Figure 7:**
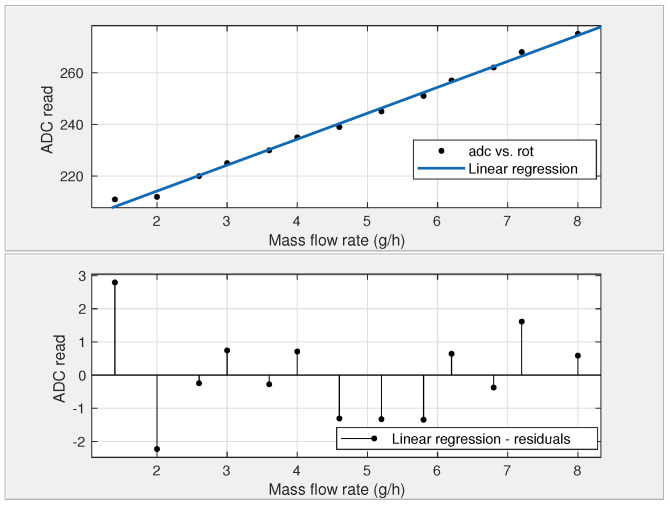
System calibration using a linear regression model. The units on the vertical axis represent ADC counts.

For the atmospheric conditions in Ouro Preto, Brazil (approximately 88 kPa at 1170 m altitude) and an average temperature of 31 °C, the specific mass of HHO is 0.152 kg/m^3^, with a volumetric composition of 86% hydrogen ($H_{2}$) and 14% water vapor ($H_{2}O$). This results in a hydrogen-rich gas range of 0 to 18 g/h for a flow range of 0 to 2000 cm^3^/min. During the flow sensor calibration, we tested a range from 0 to 8 g/h. Figure 7 presents the data from multiple experiments measuring the mass flow rate using a rotameter and the analog-to-digital (ADC) readings from the F1012 sensor. The figure also displays the linear regression analysis of this data, represented by the equation

(4) $$\dot{m}=0.099265\;\cdot \;ADC-19.254,$$

where $\dot{m}$ represents the sensor flow rate and ADC is the analog-to-digital read. The calibration linear regression model obtained an Euclidean linear norm of residuals of 0.4742 and a determination coefficient ($R^{2}$) of 0.9957. Despite the narrowed range, the regression analysis showed a high determination coefficient and a low Euclidean linear norm of residuals. These findings indicate that the cost-effective sensor is suitable for this specific application.

**Figure 8:**
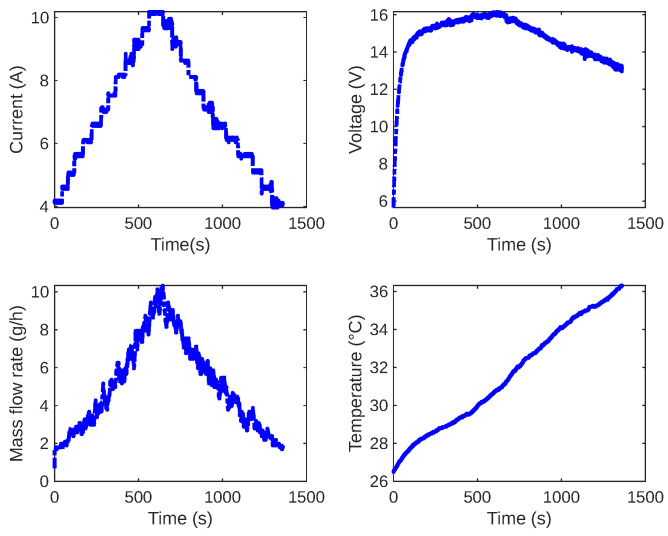
Measured variables by the instrumentation system. The superior plots from left to right show the current and voltage, respectively. The inferior plots show the mass flow and the electrolyzer temperature.

After the flow sensor calibration, we experimented to adjust the continuous electric current from the bench source. We increased it from 4 A to 10 A and then decreased it from 10 A to 4 A, with a step of 1 A. The adjustments were made every 100 s on average. This experiment was useful for observing the mass flow rate behavior on incremental and decremental steps. Furthermore, this experiment aimed to collect and analyze data on the electrolytic cell’s electric current, voltage, flow, and temperature. Figure 8 displays the measured variable values over time.

### 5.2. Controller Design and Application

**Figure 9:**
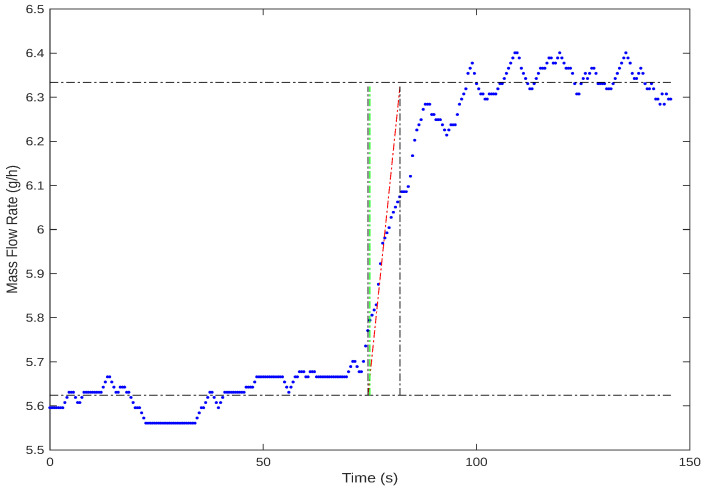
Process Reaction Curve method. The mass flow rate is indicated by the blue signal, the green line marks the step instant, and the red line denotes the tangent at the inflection point.

As a first step, we began with the model identification of the inner dynamic of the system, which is represented as $G_{2}$. And later, after closing the inner loop, we identified the model that represents the dynamic of $G_{1}$. We chose the Process Reaction Curve method to identify both models for simplicity. Figure 9 illustrates the identification model being applied for identifying $G_{1}$, where its output is the gas flow rate measured by the sensor. The identified models served as the base for the computation of controller parameters. The models structures were assumed to be the first order plus time delay, which was decided by visualizing the step responses. And finally, the model parameters were calculated as

(5) $$G_{2}\left(s\right)=\frac{0.12}{1.44s+1},\mathrm{and}G_{1}\left(s\right)=\frac{0.44}{6.50s+1}.$$

Once the parameters representing the dynamics of the processes were identified, we proceeded to the next step: tuning the PI controllers. We opted for the Simple Internal Model Control (SIMC) technique [40,41] due to its simplicity and the flexibility it offers in selecting the tuning parameter λ. This parameter allows us to specify the smoothness or aggressiveness of the controller’s response, ensuring the process variable stays as close to the reference as possible. The following transfer functions show the tuned parameters for both controllers:

(6) $$C_{2}\left(s\right)=2+\frac{5}{s},\mathrm{and}C_{1}\left(s\right)=2.25+\frac{0.34}{s}.$$

**Figure 10:**
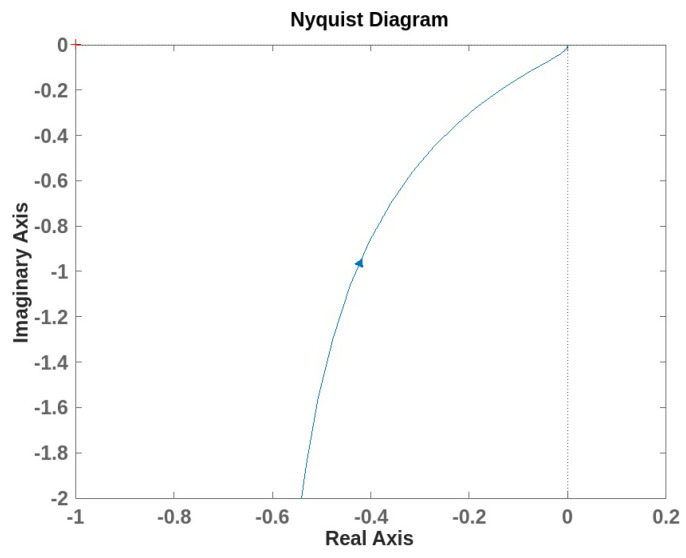
Nyquist plot for the open inner loop with maximum sensitivity $M_{s1}$ equals 1.28.

**Figure 11:**
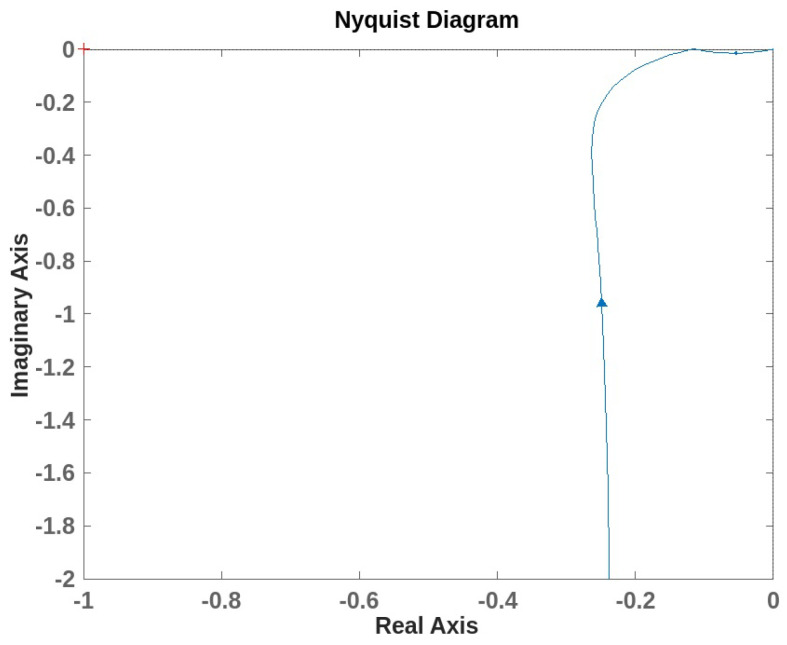
Nyquist plot for the open outer loop with maximum sensitivity $M_{s2}$ equals 1.17.

We defined the transfer functions, and we evaluated the system stability using the Nyquist diagram for both the inner and outer loops. Figure 10 and Figure 11 show the Nyquist plots for each loop. In both cases, the plots do not encircle the critical point −1+j0, which suggests that there are no right-half-plane poles in the closed-loop system of the loops. The distance from the critical point to the Nyquist plot is indicative of stability margins. The plot is sufficiently away from −1, suggesting stability. Moreover, the maximum sensitivity function was used to measure the system’s robustness. We obtained $M_{s1}$ equals 1.3 for the inner loop and $M_{s2}$ equals 1.2 for the outer loop. According to Åström and Hägglund [43], the range of 1.2 to 2 is a reasonable trade-off between performance and robustness.

**Figure 12:**
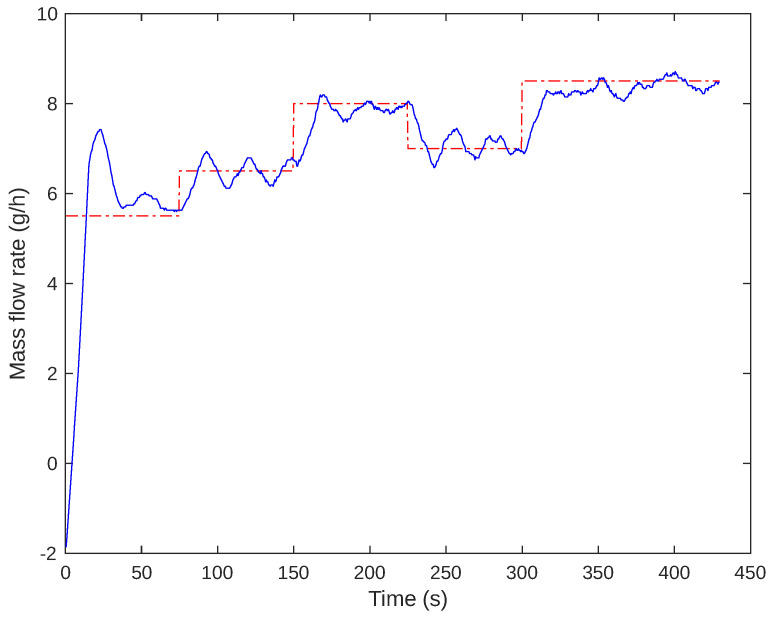
Gas flow control behavior. The set-point is shown in red, while the measured values are in blue.

**Figure 13:**
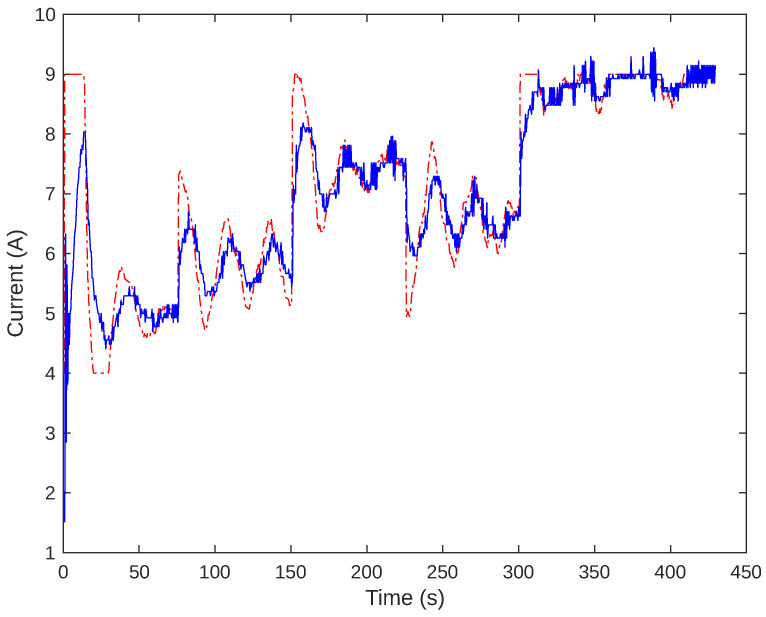
Current control behavior. The set-point is shown in red, while the measured values are in blue.

The controllers designed were integrated into the firmware of the Arduino PI control system. The script calculates control actions based on sampled measurements taken at regular intervals. An experiment was conducted to evaluate the cascade control behavior by adjusting the system set-point five times: 5.5, 6.5, 8.0, 7.0, and 8.5 g/h. This experiment is illustrated in Figure 12 and Figure 13. Figure 12 displays the modified set-point for the flow controller and the measured mass flow. Conversely, Figure 13 shows the outcome of the slave process, indicating the current set-point and measured values. The system’s performance with various set-points was observed to analyze if the system response aligns with the desired values. For the 5.5 g/h set-point, an overshoot of around 32% was noted. Over time, after 150 s, there was a more significant convergence towards the cell flow set-point, reaching the projected controller limit by 840 s.

The controller tuning was conducted within the system’s operational range of interest. Typically, the open-loop system is transitioned to the operating region, and feedback control is activated. The initial set-point, which starts from zero and goes up to 5.5 g/h, is expected to have some overshoot. However, this problem was eliminated after the first set-point transition.

**Figure 14:**
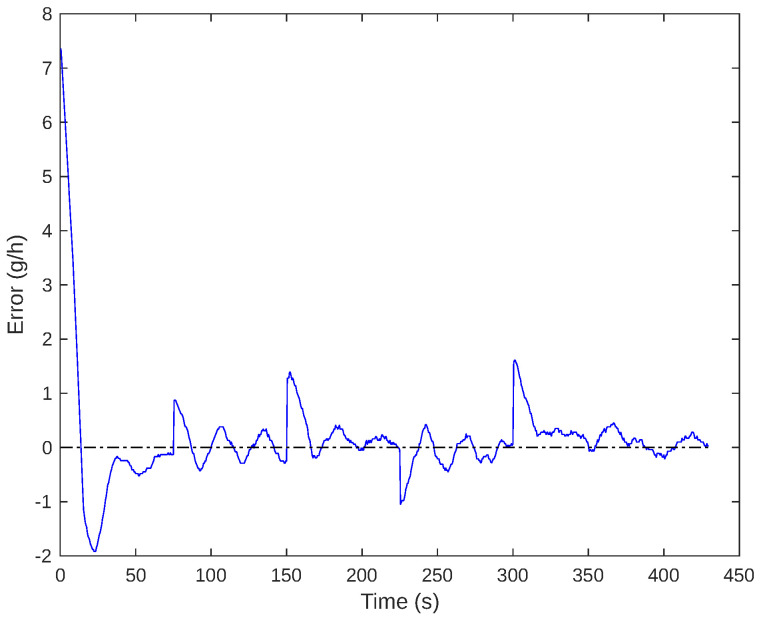
The error curve during the experiment with set-point variation.

During the gas flow controller test, errors were measured as depicted in Figure 14. It was noted that the error was consistently less than 0.5 g/h in all cases when the system was in a steady state, with a 5% accommodation criterion.

## 6. Conclusions

This study presented a cost-effective instrumentation and control system for an alkaline electrolyzer. To assess the system’s performance, we analyzed data gathered over time and observed the system’s dynamic during the experiment.

To measure hydrogen production, we calibrated a gas microflow sensor (Winsen F1012) using a linear regression approach. We achieved a determination coefficient ($R^{2}$) of 0.9957. Our study shows that the cost-effective F1012 sensor is appropriate for measuring electrolysis gas and was successfully employed as feedback in a flow cascade control.

We developed a PI control system to regulate electrolysis gas production based on the current applied to the system. This system replaced the manual electric current control of the Instruterm source with an Arduino and computer interface. The cascade control system was designed to regulate the mass flow rate of the electrolytic cell based on electric current control.

Experiments evaluating the cascade method’s effectiveness revealed an error rate of 0.5 g/h during steady-state operation, showcasing the control structure’s efficiency in managing hydrogen flow in electrolysis. Subsequent projects will explore the use of hydrogen-rich gas as an additive in truck diesel engines and control strategies based on engine speed. Additionally, we aim to compare the proposed technique with a model predictive control approach to assess its relative performance.

## Figures and Tables

**Table 1:** Comparison of the related works and our work.

Study	PI Control	Cascade Strategy	Cost Effective	Alkaline Electrolyzer	Gas Flow Sensing
Bobadilla et al. (2018) [12]	✗	✗	✗	✓	✓
Bobadilla et al. (2018) [13]	✗	✗	✗	✓	✓
Baltacıoglu (2018) [19]	✗	✗	✓	✓	✓
Figueiredo et al. (2018) [20]	✗	✗	✓	✓	✗
Flamm et al. (2021) [21]	✗	✗	✓	✗	✓
Huang et al. (2022) [22]	✗	✗	✓	✓	✗
Makineni et al. (2022) [23]	✓	✓	✓	✗	✗
Ruomei et al. (2023) [24]	✗	✗	✗	✓	✗
Folgado et al. (2022) [25]	✗	✗	✗	✗	✓
Folgado et al. (2023) [26]	✗	✗	✗	✗	✓
David et al. (2021) [27]	✗	✗	✗	✓	✗
Our study	✓	✓	✓	✓	✓

## Data Availability

Data are contained within the article.
